# Programmed Death-Ligand-1 Expression in Non-Small Cell Lung Cancer and Prognosis

**DOI:** 10.4274/balkanmedj.galenos.2018.2018.0392

**Published:** 2019-05-10

**Authors:** Songül Şahin, Şebnem Batur, Övgü Aydın, Tülin Öztürk, Akif Turna, Büge Öz

**Affiliations:** 1 Pathology Laboratory, Çankırı State Hospital, Çankırı, Turkey; 2 Department of Pathology, İstanbul University-Cerrahpaşa, Cerrahpaşa School of Medicine, İstanbul, Turkey; 3 Department of Thoracic Surgery, İstanbul University-Cerrahpaşa, Cerrahpaşa School of Medicine, İstanbul, Turkey

**Keywords:** Lung carcinomas, non-small-cell, programmed death-ligand 1, prognosis

## Abstract

**Background:**

Prognostic significance of the programmed death-ligand-1 status in non-small cell lung carcinoma remains controversial

**Aims:**

To show the programmed death-ligand-1 expression status in patients with non-small cell lung carcinoma and its effect on the prognosis and the relationship with clinicopathologic data.

**Study Design:**

Retrospective cross-sectional study.

**Methods:**

The study included 208 cases who were diagnosed with NSCLC and who underwent surgical resection between 2001 and 2012. Programmed death-ligand-1 (SP142 clone) was applied to the histological sections acquired from the microarray paraffin blocks with immunohistochemistry. Staining intensity was scored as weak (+, 1), moderate (++, 2), and strong (+++, 3). Percentage (0%-100%) was multiplied by staining intensity (1-2-3) to calculate the H score. Four different cut-off values were used; 1: ≥1% (independent of intensity), 2: ≥5% (independent of intensity), 3: ≥5% moderate/strong staining (except for weak staining), 4: H score ≥30 values were considered positive. In this study, staining a single cell at any intensity was considered positive.

**Results:**

Thirty-four out 208 cases (16.3%) had PDL-1 positive staining. PDL-1 expression was observed in patients with non-small cell lung carcinoma independent of the histological type or subtype (range; 0-25%). When the cut-off level was set to ≥5% with moderate and strong staining, the median overall survival was 45 months for the PD-L1 positive group and not reached for the PD-L1 negative group (p-value 0.024). PD-L1 positivity was significantly higher in patients over the age of 60 years and in cases with a tumor diameter of more than 5 cm (p=0.023 and 0.025, respectively).

**Conclusion:**

PD-L1 expression is positive in 16.3% of patients with non-small cell lung cancer and may have a negative prognostic value.

Among all types of cancer, lung cancer has the highest death rate in men and women (1). Treatment selection for patients with lung cancer is based on the histology type, tumor molecular characteristics, tumor stage, and the patient’s performance status. Survival rates remain low although recent improvements have been made using multimodal treatments and targeted therapies (2). New studies are being conducted on lung cancer related to tumor immunotherapy (3). Long-term responses have started to be achieved with monoclonal antibodies, which target the immune system checkpoints (check-point inhibitors) (4).

Effective immunity against cancer is dependent on the compatibility of cytotoxic T lymphocytes activity, which is related to the balance of negative and positive signals. CD28 and inducible T cell co-stimulator are positive co-stimulatory and they provide T cell activation and proliferation by binding to the ligand from the B7 family. Programmed death-ligand (PDL)-1 and PD-L2 are members of the B7 family. On the other hand, there are negative regulatory molecules on the cell surface that inhibit T cell activation or prompt apoptosis. These decrease the T cell activation by binding to the PD-1 receptors. This is an important step for the immune response to prevent tissue damage caused by induced inflammation. However, in cancer cells, PD-L1 and PD-L2 suppress the T cell attack and provide an escape from the immune system. Therefore, the tumor cells can form an appropriate tumor microenvironment and continue proliferation (5).

PD-L1 and PD-L2 expression have been shown in activated T cells, B cells, macrophages, dendritic cells, thymus endothelium, heart, and placenta. In addition, PD-L1 expression was shown in lung, ovary, breast, glioblastoma, head and neck carcinomas (6).

Previous studies have shown that prognosis is worse in tumors with PD-L1 expression compared to those without PD-L1 expression (7,8). The monoclonal antibodies that inhibit the PD-1/PD-L1 pathway also abolish the tumor cell inhibitory effect on the immune system. Immunohistochemically, it was shown that the response rate to the treatment with this monoclonal antibody in tumors with PD-L1 expression is higher. Besides its significance as a negative prognostic factor, PD-L1 expression in the tumor is important as a predictive biomarker for therapies targeting this molecule (9).

Therefore, the aim of the present study was to evaluate PD-L1 expression and its effect on the prognosis and the relationship with clinicopathologic data in patients with non-small cell lung carcinoma (NSCLC).

## MATERIALS AND METHODS

The study was approved by the İstanbul University-Cerrahpaşa, Cerrahpaşa School of Medicine ethics committee and was carried out according to the ethical principles of the Helsinki Declaration. The informed consent form was taken from patients. The study included 208 cases who were diagnosed with NSCLC and who underwent surgical resection between January 1, 2001, and December 31, 2012. Surgical procedure and stage information were retrieved from the Department of Thoracic Surgery database. Survival data were obtained by contacting 113 patients via telephone.

Microarrays of 4 mm punches were taken from the tumor blocks for the immunohistochemical study. The areas surrounded by inflammatory cell infiltration that best represent the tumor were selected. Immunohistochemical staining was performed using an automatic device (BenchMark XT IHK/ISH Staining Module, Ventana Medical Systems Ins., Medical Systems, Tucson, AZ, USA). Sections were obtained from the 10% paraffin blocks. Deparaffinization was performed using solutions and they were rehydrated using a series of decreasing alcohol concentrations. Samples were kept in 10 mmol/L buffered citrate solution for 30 minutes at 36 °C. Afterward, primary antibody PD-L1 [1/25 dilution, 32 minutes incubation, monoclonal, SP142 clone, Spring Bioscience (Spring) Roche/Genentech] antibody was applied to the slides.

### Immunohistochemical evaluation

The placenta was used as the control tissue and percentage rates were given. Tumor cells that show membranous staining were counted out of at least 100 tumor cells. Staining intensity was scored as weak (+, 1), moderate (++, 2), and strong (+++, 3). Percentage (0%-100%) was multiplied by staining intensity (1-2-3) to calculate the H score. H scores were between 0 and 300.

Four different cut-off values were used; 1: ≥1% (independent of intensity), 2: ≥5% (independent of intensity), 3: ≥5% moderate/strong staining (except for weak staining), 4: H score ≥30 values were considered positive.

Additionally, the staining in tumor-infiltrating lymphocytes (TIL) or peritumoral inflammatory cells was also recorded.

### Statistical evaluation

Fisher’s chi-square test and Pearson’s chi-square test were used in the comparison of categorical data and Mann-Whitney U test was used in the parameters comparison between groups. Kaplan-Meier analysis was used to examine the effect of PD-L1 positivity on mortality and survival rates. The statistical analysis was performed using the SPSS 21.0 statistical software package. The results were evaluated at a 95% confidence interval and p<0.05 significance level.

## RESULTS

From the 208 cases, 88.5% were male (n=184) and 11.5% were female (n=24). The average age was 60 (range 39-80). Data regarding the clinical and pathological characteristics of the cases are presented in Table 1. Survival data were available for 184 out of 208 cases; 31 (16, 8%) of these cases were not included in the survival analysis because they died within 2 months after the surgery. Of the remaining 153 (83.2%) patients, 37 (20%) died and 116 (63%) survived. The median overall survival time was 30 months (3-142). The median survival time of death cases was 24 months (3-76). The median survival time of the survivor cases was 33 months (4-142).

### Immunohistochemical findings

**Figure 1:**
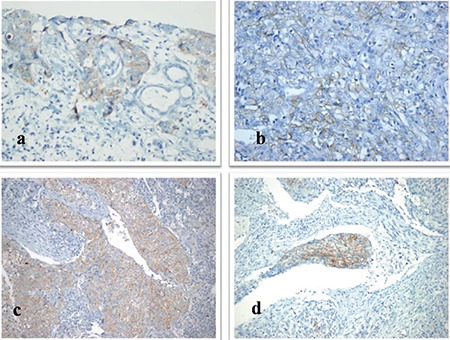
a-d. Immunohistochemistry programmed death-ligand-1 antibody staining at different rates and intensity in tumor cells. 20% wear staining (immunohistochemistry, ×400) (a), 75% moderate staining (immunohistochemistry, ×400) (b), 90% strong staining (immunohistochemistry, ×100) (c), 30% strong staining (immunohistochemistry, ×200) (d).

**Figure 2:**
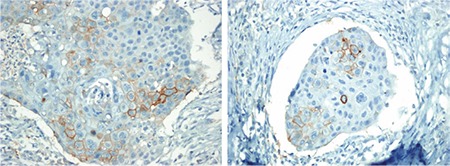
Heterogeneous programmed death-ligand-1 antibody staining in the same tumor (immunohistochemistry, ×400).

Membranous staining was observed in 34 cases (16.3%) at various rates and intensities. Staining was observed in 2 cases at 90%, 1 case at 80%, 1 case at 75%, 1 case at 70%, 2 cases at 60%, 1 case at 50%, 1 case at 40%, 2 cases at 30%, 3 cases at 20%, 8 cases at 10%, 2 cases at 5%, 1 case at 4%, 7 cases at 1%, and 2 cases at less than 1% (Figure 1). Staining intensity varied according to the tumor areas examined. In some cases, tumor cells in one area showed strong membranous staining, whereas the neighboring tumor cells showed weak positivity (Figure 2). PD-L1 staining rates observed in the tumors are presented in Table 2.

Among the cases with adenocarcinoma (AC) showing PD-L1 expression, a solid pattern was predominant in 5 and a lepidic pattern was predominant in 2 cases. Four cases (1.9%) showed staining with PD-L1 antibody in type 2 pneumocytes. Various rates of staining were observed in the TILs in 38 (18.27%) cases. While no staining was observed in the tumor cells of 11 (28.9%) cases, various rates of staining were observed in the tumor tissue of 27 cases (71.1%).

Positivity rates of cases showing PD-L1 expression varied when different cut-off values were used. Thirty-two cases (15.4%) were categorized as positive staining with a cut-off value of ≥1%, 24 (11.5%) cases were categorized as positive staining with a cut-off value of ≥5%, 19 cases (9.1%) were categorized as positive staining with a cut-off value of ≥5% with moderate or strong staining, and 12 cases (5.8%) were categorized as positive staining when the H score was ≥30.

Using a cut-off level of ≥5%, the rate of positivity was 8.4% (9/107) in squamous cell carcinoma, 8.3% (6/72) in AC, 25% (2/8) in large cell neuroendocrine carcinoma, 25% (1/4) in large cell carcinoma, and 20% (1/5) in pleomorphic carcinoma. The overall rate of positivity was 9.1%. When the clinical parameters were evaluated according to different cut-off values, the mild stromal response was higher than the moderate and intense stromal response in cases with moderate and strong ≥5% staining (p=0.019). Similarly, mild inflammation accompanying tumor was significantly higher compared to moderate and severe inflammation (p=0.041) (Table 3).

**Figure 3:**
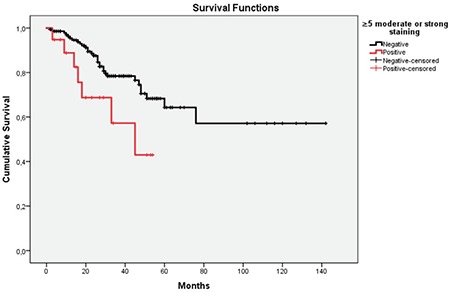
Survival times between programmed death-ligand-1 negative when cut-off value is ≥5% moderate or strong straining. The median overall survival was 45 months for the PD-L1 positive group and not reached for the PD-L1 negative group (p-value 0.024).

Independent from the cut-off level used, there was a positive correlation between PD-L1 positivity in the TILs and PD-L1 positivity in the tumor cell (p=0.00). When the cut-off level was set to ≥5% with moderate and strong staining, the median survival was 45 months (standard error: 13.752, confidence interval: 18.047-71.953) in PD-L1-positive cases. According to the Kaplan-Meier analysis, the difference in survival times between PD-L1-positive and PD-L1-negative cases was significant. Survival advantage conferred by PD-L1 negativity was demonstrated in statistical terms (log-rank p=0.024; (Figure 3).

PD-L1 positivity was significantly higher in cases with a tumor diameter of more than 5 cm when compared to cases with a tumor diameter less than 5 cm (p=0.025). When the comparison was made with the cut-off level to ≥5% and ≥1%, PD-L1 positivity was significantly higher in patients over the age of 60 years when compared to patients under the age of 60 years (p=0.023 and p=0.015, respectively).

There was no relationship between PD-L1 expression and other clinicopathologic data (gender, diagnosis, tumor subtype, tumor grade, smoking, pathologic stage, clinical stage, pleura invasion, lymphatic, vascular, perineural invasion, lymph node metastasis status, or necrosis).

## DISCUSSION

Immunotherapy has become the new treatment option in many malignancies. Observation of effective treatment responses particularly in malign melanoma and renal cell carcinoma has accelerated the studies regarding the applicability of immunotherapy in lung cancer (3,10).

With the discovery of PD-1/PD-L1 receptors and its interaction between the tumor cell and the immune system, studies have shown how the tumor blocks the immune system and progresses using this receptor signaling (11). PD-L1 is a transmembrane protein with a cytoplasmic tail. Membranous or cytoplasmic staining can be observed according to the binding point of the PD-L1 antibody. Cytoplasmic staining was shown with quantitative immunofluorescence staining and membranous or cytoplasmic staining can be observed in IMC according to the tumor type and the antibody used. The studies on the localization of PD-L1 staining in tumors have shown a predominant membranous/perinuclear staining in melanoma and membranous staining in NSCLC (11,12,13,14). It is suggested that membranous staining pattern should be considered for the PD-L1 SP142 clone used in the present study (15). Cytoplasmic staining was not observed in our cases.

Different cut-off values were used in the literature to evaluate immunohistochemical PD-L1 expression. Only some studies have used the extensiveness of staining. There are also studies that used modified methods besides the H score where the extensiveness of staining and staining intensity are evaluated together (6,7,8,12,13,16,17,18). We used four different cut-off values in our study (independent of intensity ≥1%, independent of intensity ≥5%, ≥5% moderate/strong staining, H score ≥30). We compared all clinical and pathologic parameters with the PD-L1 results we acquired with these cut-off values. Among the clinical data, we observed a difference in terms of age and survival, whereas no difference was observed in pathological data. In a meta-analysis conducted on this subject, it was found that even 1% staining with PD-L1 antibody could be sufficient to have a predictive value. Some studies suggest that indicating the absence or presence of staining would be enough, different cut-off levels while using complex systems may cause intra and interobserver variability (19). Martinez Marti et al. (20) compared their results using different cut-off values. The cut-off values were ≥5%, ≥1% and >1 H score and the results show high compatibility with each other.

The rate of PD-L1 expression reported in the literature varies depending on the cut-off level used or localization of staining (cytoplasmic and/or membranous). This rate ranges from 7.4% to 72.7% (7,16,17,18). In our study, staining a single cell at any intensity was considered positive and 34 (16.3%) out of 208 cases were found to be PD-L1 positive. When the cut-off level was set to ≥5% moderate and strong staining, the rate of PD-L1 positivity was found to be 9.1%. When the survival analysis was carried out over this value, an inverse relationship was found between PD-L1 expression and survival. Independent of its predictive value, some studies have suggested that PD-L1 expression has a prognostic significance. The prognosis is worse in patients with PD-L1 expression compared to those without PD-L1 expression and PD-L1 can be used as a negative prognostic factor (7,8,11,21). In our study, when PD-L1 positivity was based on a cut-off value of ≥5% moderate and strong staining, the survival time of PD-L1-positive patients was shorter than PDL-1-negative cases. There are also studies showing that there is no relationship between PD-L1 expression and prognosis, or reporting that patients with PD-L1 expression survive longer (13,16,22). A poor prognosis is expected in cases of anti-tumoral suppression response by PD-L1 (23).

When comparing small biopsy and resection materials, Kitazono et al. (24) showed that PD-L1 results in both materials showing 92% concordance. In our study, microarray paraffin block was prepared by choosing 4 mm of the tumor from the resection materials. While evaluating tumor cells PD-L1 expression, it was observed that the extent and intensity of staining varied across the areas. When the heterogeneous structure of NSCLC is considered, immunohistochemical evaluation in small biopsy samples may not reflect the entire tumor tissue.

Many studies evaluating the relationship between PD-L1 expression with age and gender have found no significant difference (7,17,20). However, PD-L1 positivity is significantly higher in women in the studies by D’Incecco et al. (6) and Azuma et al. (11) and in young patients in the study by Cooper et al. (16). In our study, when the cut-off level was set at ≥5% or ≥1%, PD-L1 expression was higher in patients over 60 years. There was no relationship between gender and PD-L1 expression. We believe that the relationship between age and PD-L1 expression may be due to the increase in both tumor burden and mutation due to age.

PD-L1 expression status can vary according to the tumor type. Schmidt et al. (18) have found higher PD-L1 expression in 321 cases with NSCLC and squamous cell carcinoma compared to other types. In their studies, Mu et al. (8) and Konishi et al. (13) detected higher PD-L1 expression in AC compared to squamous cell carcinoma. In our study, PD-L1 expression at any intensity was higher in squamous cell carcinoma (19.6%, 21/107) compared to AC (12.5%, 9/72), but there was no statistically significant difference. Similar results exist in the literature (16).

There are studies that have addressed histological pattern and invasion status together with PD-L1 expressions in AC. In their study, Zhang et al. (7) observed higher PD-L1 expression rates in solid AC compared to those in minimally invasive AC and AC in situ, and it was interpreted that PD-L1 expression can increase depending on the invasion status and tumor aggressiveness. Micro-invasive AC or AC in situ was not included in our study and staining was detected in cases with solid AC and lepidic pattern adenocarcinoma. There was no relationship between PD-L1 expression and AC patterns.

Sarcomatoid carcinomas are poorly differentiated tumors compared to other NSCLCs and have a poor clinical course. In the study by Velcheti et al. (25), the rate of PD-L1 expression was 69.2% in 13 cases with sarcomatoid carcinoma among 458 cases with NSCLC, and this rate was found to be 27.4% in other histological subtypes. Similarly, in the study by Kim et al. (26), the rate of PD-L1 expression in 41 cases with pleomorphic carcinoma was 90% (37/41) and more positivity was reported in the sarcomatoid regions compared to regions of differentiated carcinoma. In our study, there were a few patients with pleomorphic carcinoma and the rate of pleomorphic carcinoma-L1 expression was 20% (1/5). This rate is higher than the overall rate of positivity. Moreover, it was observed that these cases had diffuse and intense staining. In addition, these patients also showed staining on the TILs. In the literature, a high rate of PD-L1 positivity in pleomorphic carcinoma or carcinosarcoma has been explained by low differentiation level in the tumor and accompanying intense inflammation. Inflammation inside and around the tumor is related to negative prognosis in sarcomatoid carcinoma. It is thought that this can also be related to the mechanisms suppressing the immune system (PD-1, PD-L1, cytokine, Treg cell, T cell co-inhibitors) (26).

Schultheis et al. (27) studied PD-L1 expression using two different clones in 94 cases with small cell carcinoma and observed no staining in the tumor cells. Cases with SCLC were not included in the present study and staining was observed in 25% (2/8) of cases with large cell neuroendocrine carcinoma. Although the number of cases is low, PD-L1 expression was detected in 25% (1/4) of large cell carcinoma cases, a finding consistent with the literature (16). Other cells accompanying the tumor were also evaluated in terms of PD-L1 expression. While staining is observed with PD-1 on TILs in many studies, different results exist with regard to PD-L1 staining (18,27,28). In two different studies, the PD-L1 expression in the tumor tissue was found to be 52% and 72%, staining rates in the parenchyma were 4.8% and 9.3%, respectively (8,21). Chen et al. (21), evaluated PD-L1 expression in 120 cases with NSCLC and 10 benign control tissues, and they observed PD-L1 expression in 57.5% of the tumors and no staining in the benign control tissues. In the study by Gettinger et al. (29), PD-L1 staining was observed on the lymphocytes and scoring was performed according to the staining percentage. Because of this study, when a relationship was detected with treatment response, it is suggested that TILs should also be evaluated along with the tumor cells.

In our study, PD-L1 staining on TILs was observed in 38 cases (18.3%) at variable rates. PD-L1 positivity in the tumor cells was higher in cases with PD-L1 positivity on the TILs. In our study, PD-L1 positivity on the TILs was observed to be parallel to the positivity in the tumor. In the evaluation of the relationship between PD-L1 expression and clinical and pathological data, Schmidt et al. (18) observed a higher rate of PD-L1 expression in cases receiving adjuvant treatment and in those with larger tumor size and lymph node metastasis. In our study, PD-L1 positivity was significantly higher in the cases with a tumor diameter larger than 5 cm.

In the studies by Yang et al. (12) and Grosso et al. (30), a positive relationship was detected between PD-L1 expression in tumor and surrounding inflammatory response. In our study, a negative correlation was observed between the peritumoral stromal, inflammatory response, and PD-L1 expression. This inconsistent result with the literature can be explained by the fact that PD-L1 expression changes in response to different stimuli and the limited number of studies on this subject. In the studies conducted with PD-L1 antibodies, the results can vary depending on many non-standardized factors such as different antibody and clone use, different cut-off values, localization of the staining in the cell, disease stage, previous treatments, use of archived or fresh tissue, and working on primary or metastatic tissue. In the literature, there is an agreement that PD-L1 expression is a negative prognostic factor and is tumor expression can be used as a biomarker in the selection of anti-PD-1/PD-L1 antibody (check-point inhibitor) treatment. The common opinion is that treatment response is higher and disease-free survival is longer in patients with PD-L1-positive tumors at any intensity (3). Clinical response was achieved also in PD-L1-negative cases, although the rate of response was lower (9). There is a need for a biomarker that would allow predicting the patients who could achieve better treatment response. Immunohistochemical evaluation of PD-L1 receptors is gaining ground as a biomarker that can be used for this purpose.

It should be noted that there may be many parameters that will affect the prognosis at the same time. Many studies to be done in the future will make it clear.

In this study, results on PD-L1 expression and its relationship with survival in NSCLC were in parallel to the literature. Moreover, it can be used as a negative prognostic factor independent from the selected treatment option.

## Figures and Tables

**Table 1:**
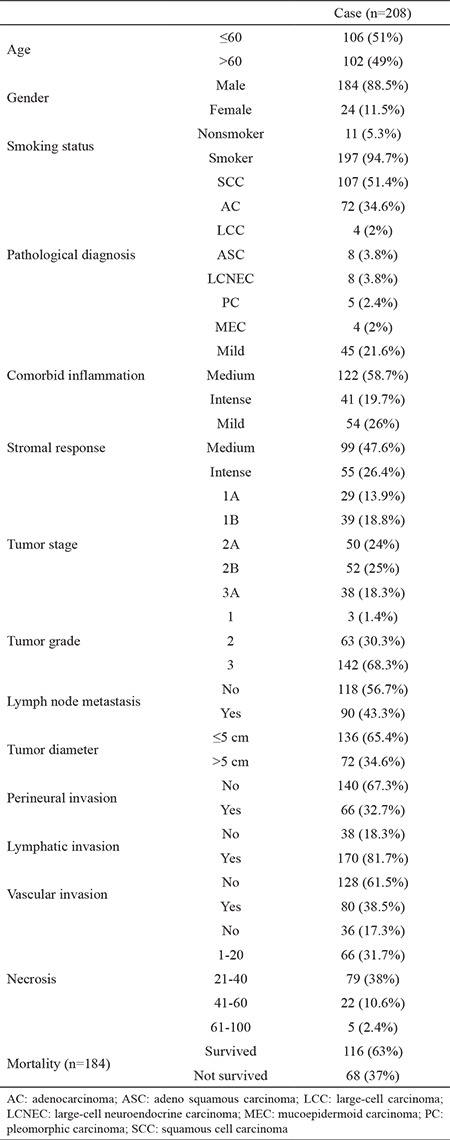
Clinical and pathological characteristics

**Table 2:**
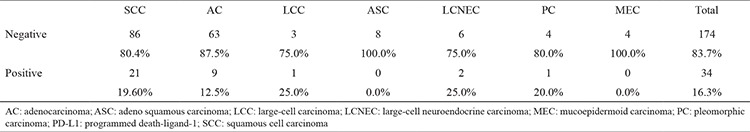
Distribution of PD-L1 staining ratios according to the diagnosis

**Table 3:**
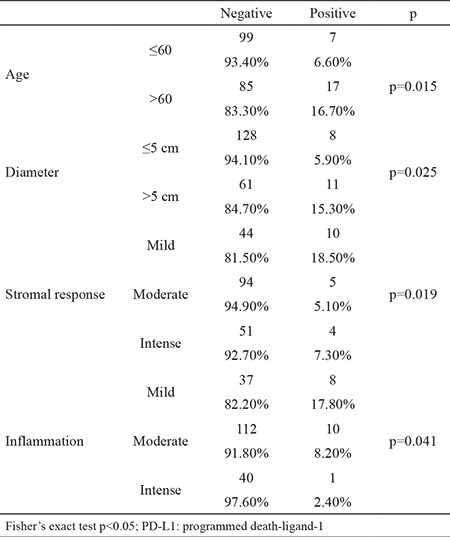
Clinicopathologic parameters associated with PD-l1
